# Public health benefits of water purification using recycled hemodialyzers in developing countries

**DOI:** 10.1038/s41598-020-68408-1

**Published:** 2020-07-06

**Authors:** Jochen G. Raimann, Joseph Marfo Boaheng, Philipp Narh, Harrison Matti, Seth Johnson, Linda Donald, Hongbin Zhang, Friedrich Port, Nathan W. Levin

**Affiliations:** 1 Easy Water for Everyone, 10 Bank Street, Suite 560, White Plains, NY 10606 USA; 2 Research Division, Renal Research Institute, New York, USA; 3 Katz School at Yeshiva University, New York, USA; 4 Easy Water for Everyone, Accra, Ghana; 5 Department of Field Epidemiology and Applied Biostatistics, Kwame Nkrumah University of Science and Technology, Kumasi, Ghana; 6 Ghana Health Services, Big Ada, Ghana; 7 Department of Epidemiology and Biostatistics, CUNY Graduate School of Public Health and Health Policy, City University of New York, New York, USA; 8 CUNY Institute for Implementation Science in Population Health, New York, USA; 9 Departments of Medicine (Nephrology) and Epidemiology, University of Michigan, Ann Arbor, MI USA

**Keywords:** Nephrology, Pathogenesis

## Abstract

In rural regions with limited resources, the provision of clean water remains challenging. The resulting high incidence of diarrhea can lead to acute kidney injury and death, particularly in the young and the old. Membrane filtration using recycled hemodialyzers allows water purification. This study quantifies the public health effects. Between 02/2018 and 12/2018, 4 villages in rural Ghana were provided with a high-volume membrane filtration device (NuFiltration). Household surveys were collected monthly with approval from Ghana Health Services. Incidence rates of diarrhea for 5-month periods before and after implementation of the device were collected and compared to corresponding rates in 4 neighboring villages not yet equipped. Data of 1,130 villagers over 10 months from the studied communities were studied. Incidence rates showed a decline following the implementation of the device from 0.18 to 0.05 cases per person-month (ppm) compared to the control villages (0.11 to 0.08 ppm). The rate ratio of 0.27 for the study villages is revised to 0.38 when considering the non-significant rate reduction in the control villages. Provision of a repurposed hemodialyzer membrane filtration device markedly improves health outcomes as measured by diarrhea incidence within rural communities.

## Introduction

Estimates from the World Health Organization and the World Bank place around 1.1 billion people in the world in a position of having to drink unsafe water. Water and sanitation, specifically access to clean water for the world population, were adopted as the Sustainable Development Goal-6 (SDG-6) by all member states of the United Nations. The deserved, widespread attention emphasizes the importance of the issue and the need for more improvement. Industrialized countries have to a large extent solved the problem and a majority of their populations has access to safe drinking water. This is mainly due to the effort of governments, strict laws, regular monitoring, efficient handling and cleaning of sewage, centralized and monitored provision of clean drinking water and lastly to a generally higher level of hygiene (including the use and provision of sanitary facilities). Due to high population growth rates, lack of economic development, and inadequate political efforts this remains a major problem in many countries with limited resources.

Rural areas in developing countries present problems of greatest magnitude. Water is still mainly carried from continually contaminated surface water such as ponds and rivers. Water is often polluted by coliform bacteria and viral pathogens. Factors such as a lack of sanitary facilities, inadequate hygiene practices and substantial flooding during rainy seasons aggravate the problem. Not only surface but also centralized, processed water are at high probability of being contaminated^1^. Wells may also be susceptible to pollution particularly when they are shallow or intermittently overcome by raising water tables. Further, in some low-income countries a flourishing business of sachet water exists, which is assumed to be safe for consumption. However, as shown in work from Nigeria these sachets are also in many cases contaminated due to improper packaging and storage, or inadequate hygiene in the processing. The incidence of diarrhea and its life threatening complications such as dehydration and acute kidney injury correlate with these factors^2^. Non-infectious contaminants in drinking water such as lead and other heavy metals, arsenic, and also organophosphates from pesticides and insecticides contribute to health hazards, problems that are not addressed with our work at present.

Since the first epidemiological studies by the physician John Snow in the nineteenth century, the deleterious effect of microbial pathogens in water has been well established. Estimates of the World Health Organization suggest that 88% of all diarrheal diseases are caused by the consumption of unsafe drinking water and the lack of adequate sanitation facilities^3^. A recent publication of the initiative has identified that a majority of cases of acute kidney injury in the developing world are (in contrast to the most frequently reported pathogenesis in first world countries) are associated with community-acquired disease and to a major part with diarrhea^4^. This is particularly evident in children 2 to 5 years of age in whom mortality is very high^5^. Overall, these data strongly corroborate why it must be a prime goal for the world community to jointly aim to achieve the SDG-6. These data provide a powerful stimulus for widespread joint action by the world community to achieve this goal.

Common approaches to counteract microbial pollution include various filtration devices: Microfiltration, ultrafiltration, nanofiltration and reverse osmosis. Membrane filtration has long been recognized as an effective and likely efficient approach to partly solve the problem in rural regions, however membranes and filtration devices are expensive, and filters are prone to clogging without proper functioning flushing methodologies. The great need that is also building the basis of the SDG-6 of the United Nations, will require an affordable solution to be made available that is not overly prone to malfunction, can sustain functionality over a long period of time and does not require too extensive maintenance in terms of parts and labor. Surface water is often polluted with parasites, bacteria and viruses that can cause serious health issues^6^. Of note, all these pathogens are larger than the pore size of the hemodialyzer that is approximately 0.003 µm. This pore size notably is smaller than most commercially available purification devices, the operation of which has been claimed to be a feasible technique for water purification^2^.

Hemodialysis is a renal replacement therapy modality that uses hemodialyzers in those suffering from renal failure to counteract the consequences of not having kidney function and to ultimately save them from dying. These hemodialyzers are mainly comprised hollow fibers in a plastic casing. This allows, after cannulation of the patient, to pass the patient’s blood inside the fibers, and along the semipermeable membrane of the fiber, until it leaves the hemodialyzer and is returned to the patient. At the same time, dialysis water, containing anions and cations in specifically defined concentrations, passes, in a countercurrent fashion, on the other side of the membrane resulting in gradient-driven diffusion allowing for toxin removal from the blood and by producing a hydrostatic pressure also removes excess water from the patient through volumetric ultrafiltration. These hemodialyzers were commonly being reused after sterilization, a practice that has changed since earlier days of dialysis and current clinical practice commonly uses hemodialyzers only once and discards them after use. Of note, this alone results in approximately 30 kg of annual waste for every (out of approximately 2 million worldwide) dialysis patient^7^. It was shown recently that used and re-sterilized hemodialyzers (a process possible at less than $2 per hemodialyzer) are effective in producing clean water from microbiologically contaminated water when pushed through these hemodialyzers under high hydrostatic pressure.

We, Easy Water for Everyone (EWfE), report here the experience and some preliminary data from the use of this relatively simple technique for preparation of drinking water from polluted river water in rural villages in Ghana that have no electricity. We provided villages with devices containing re-sterilized hemodialyzers uniquely repurposed from their hemodialysis past, which are capable of producing large volumes of water (up to 500 L/h) free of bacteria and viruses for domestic use. Here we report public health outcomes based on prospectively collected self-reported public health information on diarrhea incidence collected before and after implementation of this device in several villages.

## Material and methods

Easy Water for Everyone (EWfE) is a 501(c)(3) non-profit, non-governmental organization (NGO) in the United States, Ghana (and with other countries in progress). With the help of local politicians and stakeholders a need for water purification in the estuary of the Volta River in Ghana was identified. For those living in this region the river is the main source for drinking water even though it is known to carry pathogens. Under the supervision of local committees and administrators, EWfE started to install and maintain a device in each of the villages. The chronological order was arbitrary and data collection was commenced on the islands around Ada Foah since 02/2018.

### Water purification method

**Figure 1:**
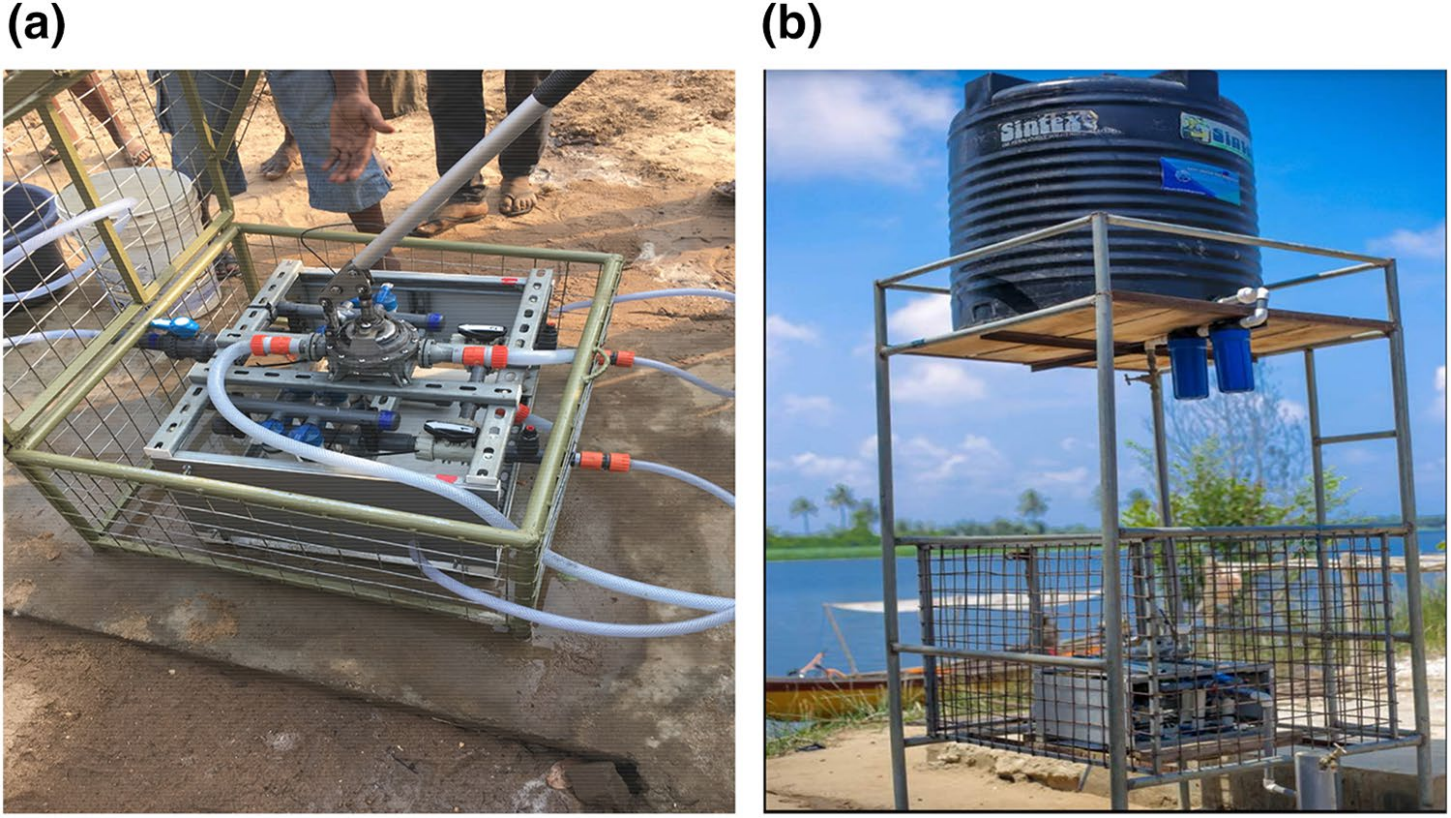
Hemodialyzer membrane filtration device used for our project. Setting with (**a**) a manual pump (up to 500 L/h) and (**b**) gravitational force (up to 250 L/h) for driving the contaminated into the re-sterilized and repurposed hemodialyzer filters.

The membrane filtration device (NUF500; NUFiltration, Israel), consists of a set of 8 hollow-fiber hemodialyzers, appropriate tubings and a faucet. These hollow fiber hemodialyzers in this project have been used as hemodialyzers once, then reprocessed and sterilized according to FDA/AAMI standards before installation into the water-purification device. Each hemodialyzer contains around 12,000 capillaries providing a membrane surface area of nearly 2 square meters per hemodialyzer. The membrane pore size is 0.003 µm, notably preventing passage of bacteria, parasites and notably also of pathogenic viruses. The output of pure water can be as high as 500 L/h when actively pumped into the device or up to 250 L/h passed into the device by gravity after being pumped into an overhead tank as used in this study. The pressure by gravity is caused by a height of about 12 feet from which the polluted water enters the eight dialyzers placed in parallel (see Fig. 1a, b).

Contaminated river water enters the inside of the capillaries (“blood” compartment) while clean water collects outside of the capillaries (“dialysate” compartment in clinical hemodialysis). Only water (and dissolved salts) passes through the pores. Organic matter that accumulates on the inside of the capillary fibers needs to be rinsed away by intermittently reversing the pressures and filtering clean water back across the membranes (backwashing) through manual pumping. It takes less than 5 min for the backflow to change from dirty to clean appearance and then regain full efficiency for providing clean water.

### Data collection

Following the approval of our research project, embedded in the non-profit endeavor, by Ghana Health Services, we initiated data collection with trained local community members to support our endeavor. Next to demographic data and water results before and after passing through the filter, we collected data monthly from the heads of households on self-reported diarrhea events in 8 villages during the months February through November 2018. This was a subset of villages served by EWfE.

In late June 2018, the hemodialyzer filtration devices became operational in 4 of these villages so that this ongoing monthly data collection started 5 months before the installation. It was concluded 5 months after the installation of the hemodialyzer filtration device. Simultaneously the same data was collected in the 4 villages without the device. For each village and each month, the count of diarrhea events and the number of persons exposed to the data collection were analyzed to estimate the monthly diarrhea incidence rates. Monthly data were summarized for each of the two groups of villages, the control group of 4 villages never exposed to the hemodialyzer water treatment and the group of 4 villages exposed to the water treatment during their second 5 months of the 10-months study period. This approach allowed comparison of the incidence rates during the first and second 5-months periods and incidence rate ratios (second/first 5 months) for the study group and the control group. Having this concomitant data allows us, in a univariate fashion, to use village populations as their own controls and consider the potential confounding effect of seasonality.

## Results

The results of water testing showed coliform bacteria at 558 CFU/100 mL in the source water (Volta River) and zero CFU in the filtrate water at the beginning of our installations in the villages of Big Ada. We studied 8 villages (4 were designated control villages and 4 were study villages) in rural Ghana. Table 1 shows the population characteristics of the study arms. Of the village populations studied in this cohort study, 11% and 8% were younger than 5 years of age and notably showed a remarkably high proportion of villagers (96% and 99%) had to resort to open defecation.

**Table 1:** Demographics of villagers in study and control villages.

	Study villages (4)	Control villages (4)
Count [villagers/villages]	441	689
Age		
< 5 years [count (%)]	50 (11.3)	56 (8.13)
5 to 18 years [count (%)]	131 (29.7)	249 (36.14)
18 to 30 years [count (%)]	103 (23.4)	130 (18.87)
30 to 50 years [count (%)]	97 (22)	151 (21.92)
> 50 years [count (%)]	50 (11.3)	103 (14.95)
Male gender [count (%)]	193 (43.76)	346 (50.22)
Sanitation facilities		
Pit latrine	18 (4.1)	1 (0.15)
Water closet	0 (0)	0 (0.00)
Public toilet	0 (0)	3 (0.44)
Open defecation	423 (95.9)	685 (99.42)

**Figure 2:**
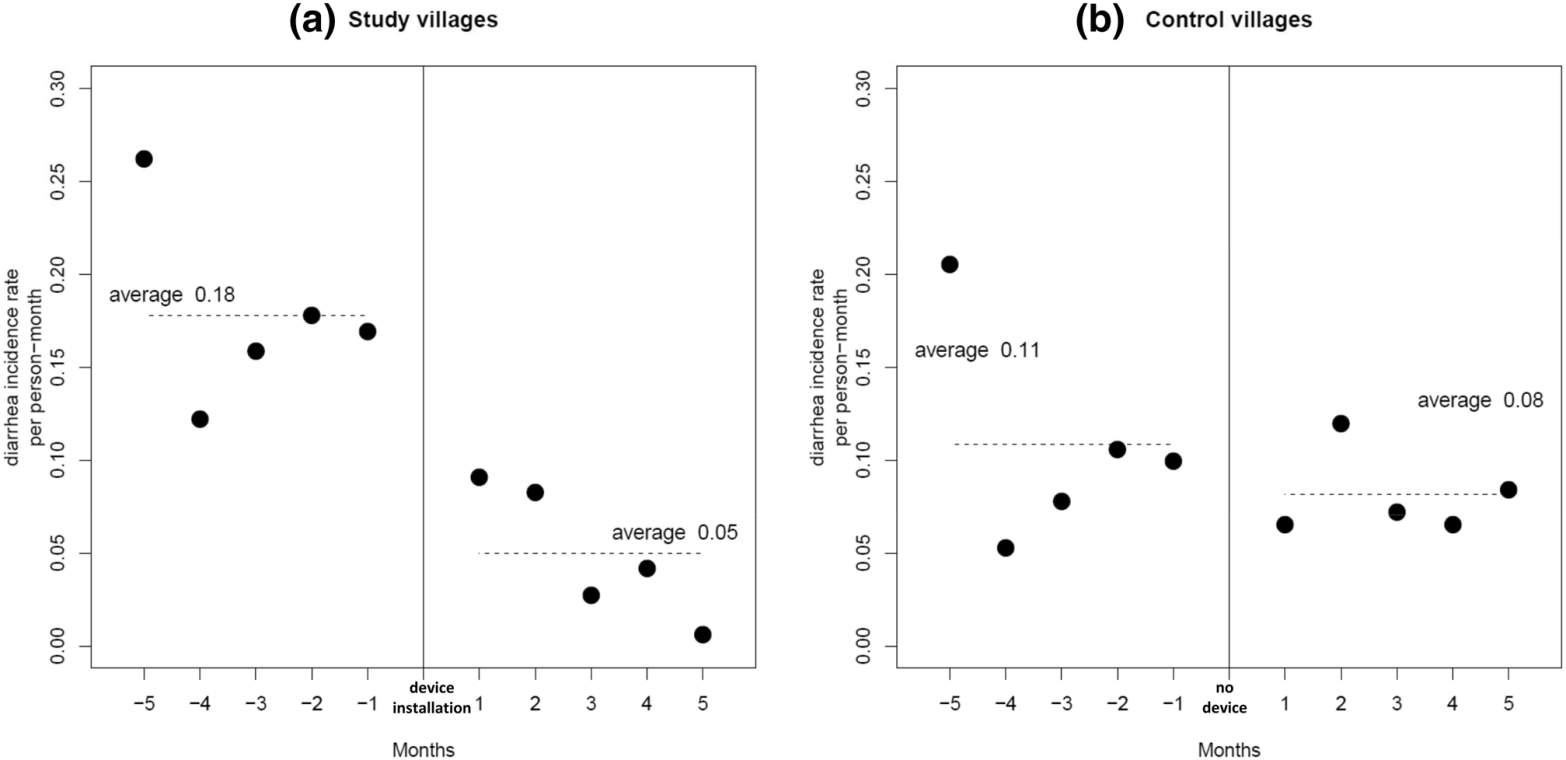
Monthly diarrhea incidence rates between February (Month − 5) and November (Month + 5) 2018 in (**a**) study villages, where the device was installed in late June 2018 and (**b**) control villages with no device installation during the same months.

Monthly diarrhea incidence rates averaged 0.18 counts per exposure month during the baseline period of the study villages and 0.11 for the same 5 months of the control group. During the first 5 months after the installation of the hemodialyzer filtration device, the rate reduced to 0.05, yielding a rate ratio for the study group of 0.28. For the control group the second 5 months gave an average rate of 0.08, showing modest non-significant reduction from the prior 5 months period with a rate ratio of 0.73 (Table 2). Figure 2a and b show the monthly data for the two periods in both village groups. The control villages of the same region and during the same calendar months allow consideration of a seasonal effect on the diarrhea incidence in the study group. Thus, using the incidence rate ratio for the second 5 months over the first 5 months gives a seasonally adjusted rate ratio of 0.38 (0.28/0.73), which translates to a diarrhea incidence rate that is reduced by 62% following initiation of the hemodialyzer filtration device in the study villages.

**Table 2:** Diarrhea incidence counts and rates for the entire 5 months A) before and B) after device implementation in the village.

	Study villages	Control villages
A) Before implementation		
Count (villagers)	357 to 441	697 to 816
Incidence count (count)	153	172
Exposure days (days)	26,208	48,272
Incidence rate (count/person-month)	0.18	0.11
B) After implementation		
Count (villagers)	312 to 359	683 to 699
Incidence count (count)	39	130
Exposure days (days)	23,576	48,748
Incidence rate (count/person-month)	0.05	0.08

## Discussion

In many countries microbiologically contaminated water is the underlying cause of gastrointestinal disease, mainly diarrhea, associated with deleterious consequences such as acute kidney injury resulting in a high mortality rate, particularly in weaned children younger than five and the elderly. Our data, collected in 4 rural communities in the Ada-East distric of Greater Accra Region in Ghana, before and after the implementation of a hemodialyzer membrane filtration device to produce clean drinking water, shows a substantially reduced risk (rate) of self-reported diarrhea by 72%. This is a major public health outcome particularly since diarrhea is well known to be associated with deleterious consequences such as acute kidney injury and death, particularly in younger children and the elderly. This finding is striking and the rigorous analytic design where each community serves as their own control allows for drawing solid conclusions. Studying and comparing our data to that of a control group which presented only with modest reduction in the incidence of diarrhea over the same time period, corroborates an effect that can be attributed to implementation of our approach. The only modest reduction of diarrhea incidence in the control villages also reduces concerns of seasonality in the incidence rates confounding our interpretation.

### Discussion of our approach in comparison with other approaches

The methods used in the present study have been effective in removing pathogens from consistently polluted river or lake water sources. During the past 3 years the on-site implementation of the hemodialyzer filtration device have allowed us to demonstrate the success of providing clean and pathogen-free drinking water to villages where the source of drinking water had been consistently contaminated. This system works well even in remote areas without requiring electricity or other external power sources. No restrictions on water use need to be imposed and use of clean water can be encouraged also for handwashing with soap. When more water is needed, the filling of the main water tank can be increased from weekly to two to three times a week (or even daily). There are several key elements that contrast our approach to other methods to produce drinking water: (1) Rejection of pathogens is highly effective and includes particles as small as pathogenic viruses, given the pore size of 0.003 µm, (2) no need to add bactericidal agents such as chlorine to kill remaining pathogens in drinking water, (3) the simplicity of this design allows its use in isolated rural villages even in areas that have no electricity, (4) this system becomes almost self-sufficient after a few villagers have been trained to do the thrice daily backwashing, (5) excellent filtration rates have been observed with this setup for over one year, (6) visits by a trained technician once or twice weekly or more frequently when necessary for refilling the large water tanks using a gas-driven pump provide some monitoring of the continued function and service and (7) relatively low cost since the reprocessed hemodialyzers are inexpensive and have shown in our 3-year experience to maintain high output rates of nearly 250 L/h (by gravity feed) for over one year. Furthermore, in circumstances where larger volumes of purified water are needed, an expanded device, employing far more dialyzers could be utilized. It would also be feasible to equip the device with solar panels which would increase water production substantially but would add to the cost.

#### Comparison of efficacy with other approaches

Attempts to purify water from microbiological contamination have been undertaken in a multitude of studies discussing purification of water from springs, boreholes, and wells, all sources with many opportunities for contamination to occur between sources and point of use. The source water is detoxified and infectious agents are reduced or removed by methods such as chlorination, membrane filtration, flocculation and others. Direct systems include conventional filtration, for example using sand through granular media which removes parasites, bacteria and possibly some viruses. Conventional filtration also includes chemical coagulants such as potassium alum added to source water which produce clots (flocs) which are in turn filtered. These processes are not easy and require expert handling by trained individuals.

Quite commonly reported is household chlorination which is a simple technique with widespread use. It improves water quality and effectively prevent diarrheal diseases. Quantity and acceptance (because of the resultant taste of the water) are downsides of this approach^5^.

With direct filtration, water passes through a medium such as sand or diatomaceous earth, a process which removes giardia lamblia, cryptosporidia, and bacteria from the water. These methods also remove color and turbidity. Filtration bags are warm bags or cartridges containing a filament to strain the water. These bags are however not useful for anything smaller than the giardia. Ceramics may be impregnated with tiny colloidal particles and allows for eradication of most bacteria and protozoan parasites. However, also this method is not adequate for virus removal. Most of these methods however are laborious, require specialized knowledge and infrastructure, and also time.

Membranes are widely used to produce safe drinking water and are the only means available to produce water free of parasites, bacteria and all pathogenic viruses.

Membranes can be divided into groups largely defined by their characteristics in regard to pore sizes. Depending on the degree of pore size, they can also produce water free of many chemical components. In the case of biologically contaminated water some membranes can produce water free of bacteria, parasites and viruses.

Hemodialyzers that are contained in the device we have chosen to implement in village structures have a semi-permeable membrane made of polysulphone and polyethersulphone. The pore size is around 0.003 µm and will not let parasites, bacteria and viruses pass, while still providing an output as large as 500 L/h.

Decreased microbial quantity in drinking water is effective in decreasing diarrhea. Effectiveness does not solely depend on the presence of improved water supplies but will also be affected by the use of sanitization facilities and handwashing with diligent soap procedures. In concert with appropriate education, these interventions will play a powerful role in improving public health outcomes. Also important in the context of effectiveness is the amount of water that is being produced over a defined period of time. In this context it is of note that our approach, even with the use of the gravitational device where water is pumped into an overhead tank and gravitation is being used to transfer contaminated water into the filter, allows for up to 250 L/h.

#### Household efforts

Household efforts include: improved water storage, chlorination, solar exposure, filtration by filter media in relationship to pore size, combined flocculation and disinfection methods. A combination of efforts including improved water supply and storage, and improved sanitation results in better water supplies thus reducing the risk of developing diarrhea. Various authors provide a range of figures for reduction of diarrhea but overall it is expected that household interventions will provide a risk reduction for diarrhea incidence^8^. The WHO promotes water treatment and safe storage of household water. Affordability, acceptability, sustainability and scale ability are all important factors and these small-scale solutions do provide improvement.

A current technology comparable to our approach are the “Aqua Towers”, an approach that also uses gravitational forces to pass water through the filter. More than 1,000 of these are active in Asia Pacific and Latin America. It utilizes ultrafiltration but the manufacturer does not reveal the membrane type. Activated carbon is used to enhance the quality of the drinking water. In addition, part of the water supply is used for hand washing. The authors claim that viruses larger than 0.01 microns are removed. However, a membrane with pore sizes as large will not exclude the rotavirus (a causative pathogen of diarrhea in up to 40% in some reported populations), and hepatitis B and C viruses, unlike the hollow fiber hemodialyzer membrane as discussed above. Of note, no outcome data have been published for the communities using the “Aqua Towers”, to the best of our knowledge.

### Strengths and limitations of our study

Surveys of diarrhea in households may be considered soft data, however the magnitude of a relative 72% reduction in the incidence of diarrhea per monitored population is strikingly large. It is also corroborated by many mothers reporting a sudden virtual absence of diarrhea in their children after availability of the hemodialyzer-filtered water. The marked reduction in the diarrhea incidence may be due to using sterile water instead of river water polluted with known pathogens, such as *E. coli*, as the main source of drinking water. Additionally, handwashing with clean water may be an important contributor to our observations. While our study cannot prove causation with certainty, the nearly stable rates in the control group suggests a causative role of the change in the water source from river water to filter-sterilized water.

Of note, we decided to not adjust for population characteristics for two reasons: the same population served as their own controls for each household and the groups of villages and secondly the incidence rates during the initial 5 months were similar for the two groups of villages.

### Further considerations beyond water purification

The effectiveness of pure drinking water, sanitation and hygiene by the Campbell/Cochrane collaboration showed 66 rigorous evaluations and 71 interventions (accounting for 30,000 children in 35 countries). Point of use water quality was associated with positive outcomes and so did hand-washing with soap. The Cochrane data base of systemic reviews discussed the effect of hand washing promotion for preventing diarrhea induced nutritional deficiency^9^, retarded child development^10^ and deaths in low- and middle-income countries. The list of interventions to improve water quality by eliminating or reducing pathogens with the objective of preventing diarrhea is substantial.

Our results on markedly reduced incidence of diarrhea after implementation of the hemodialyzer filtration device agree with prior studies. In Clasen’s data synthesis paper^11^ on 42 studies in 21 countries showed that all interventions to improve the microbial quality of drinking water were effective in reducing diarrheal incidents even though variations in design and application of water cleansing systems limit comparability of their cited studies. Results are less consistent for the role of other common environmental interventions (such as sanitation, or instruction in hygiene)^12^.

## Conclusion

Our study using monthly surveys of diarrhea in households may be considered soft data, however the magnitude of a relative 72% reduction in the incidence of diarrhea per monitored population is strikingly large. It is also corroborated by many mothers reporting spontaneously a sudden virtual absence of diarrhea in their children after availability of the dialyzer-filtered water. The marked reduction in the diarrhea incidence is likely due to using sterile water instead of using river water polluted with known pathogens, such as *E. coli*, as the main source of drinking water. It may be expected that combination of installing a membrane filtration device and combining it with WASH initiatives will have a strong amplified effect as compared to clean water provision alone. This however remains to be shown in further prospective research.

The hemodialyzer membrane filtration device used in this study was clearly associated with a substantial reduction in the incidence of self-reported diarrhea compared to the prior period and compared to a control group without the device. Use of repurposed hemodialyzers, that had already saved lives once in their initial purpose in renal replacement therapy, can again serve as an affordable means of water purification to again save lives within entire communities. Our hemodialyzer membrane filtration approach using hollow fibers with pore size as tight as 0.003 µm in the a surface-maximizing configuration used in the technology of the device described in this paper is highly effective and unique. This renders it not only eligible but potentially highly effective to allow the world population to successfully accomplish the United Nations’ Sustainable Development Goal 6.
